# Staring briefly at the sun: reflections on hope, grief and nursing

**DOI:** 10.1177/17449871261438350

**Published:** 2026-04-13

**Authors:** Pernilla Garmy

**Affiliations:** Professor, Kristianstad University, Sweden

When is it reasonable to tell your children that you have a terminal illness? Should it be before or after the Swedish Friday evening family ritual of *fredagsmys?* These are some of the reflections raised by Jenny Aronsen (2026) in her powerful paper about living with ALS. Aronsen is both a nurse and a researcher, and she herself has been living with ALS for several years. Her text is deeply moving and, I hope, will be used in many contexts, not least in nursing education.

Aronsen highlights the importance of hope. Even in situations where the outlook appears very dark, people need permission to hope. As nurses, we may have an important role in supporting and protecting that hope. This is not about denying reality, but about acknowledging the human need to find meaning, connection and possibility even in the face of life-limiting illness.

Barbara Dossey’s holistic nursing theory reminds us that nursing care must address the whole person. At the same time, it also emphasises that caregivers themselves must be attentive to their own inner lives. As nurses, we need to strengthen our capacities for presence, reflection and self-awareness, and we must dare to encounter thoughts about death and mortality (Garmy et al., 2021).

Research by Garnow et al. (2024) shows that adolescents experience existential loneliness as painful, yet also as something that can be understood as part of life and even as a potential source of personal growth. At the same time, young people describe the need for respite from existential loneliness. Remaining with these reflections for too long can become overwhelming. Their findings echo the ideas of Irving D. Yalom.

Yalom (2009) argued that existential thoughts about our own death can only be approached briefly and intermittently. He compared it to staring at the sun: we cannot endure looking for too long. The sun is powerful and beautiful, but if we stare at it continuously we become blinded. We must occasionally look away. In *Staring at the Sun*, Yalom (2009: 121) wrote that existential isolation is ‘more profound and stems from the unbridgeable gap between the individual and other people. This gap is a consequence not only of our having been thrown alone into existence and having to exit alone, but derives from the fact that each of us inhabits a world fully known only to ourselves’.

In this sense, we are ultimately alone. We enter the world alone and we leave it alone. This may sound harsh. At the same time, human life is deeply relational. A newborn child would not survive if left alone, and throughout life we constantly meet, shape and support one another. Yet experiences of grief, whether the grief associated with one’s own approaching death or the loss of a loved one, can confront us with our own existential loneliness.

In this issue, El Gharbawi et al. (2026) explore school nurses’ experiences of supporting children in grief. Their findings show that the majority of school nurses (90%) felt secure in their professional role, and most (93%) had experience of meeting children who were grieving. The qualitative findings highlighted how school nurses strive to create a sense of security for children experiencing grief. This involves both a sense of professional security for the nurse and the ability to create a supportive environment where the child feels safe and cared for. The study highlights the important role school nurses can play in creating a safe and supportive environment for grieving children in the school setting.

Another contribution in this issue focuses on supportive interventions in palliative care. Saragih et al. (2026) demonstrate that mindfulness-based interventions may reduce anxiety and depression among patients receiving palliative care, suggesting their potential value as supportive approaches for improving quality of life in difficult circumstances.

Taken together, the papers in this issue remind us that nursing is not only about managing symptoms or delivering treatments but also about accompanying people in some of the most vulnerable moments of life, moments when hope, loneliness, grief and meaning come into focus. As nurses, we may not always have answers. But we can offer presence, attentiveness and care.
